# Commentary on recent spinal cord stimulation publications

**DOI:** 10.1016/j.inpm.2023.100241

**Published:** 2023-03-17

**Authors:** David Sherwood, W. Evan Rivers, Christine Hunt, Zach McCormick, Jose de Andres, Byron J. Schneider

**Affiliations:** University Health - Lakewood Medical Center, Department of Orthopedics, 7900 Lee's Summit Rd, Kansas City, MO, 64139, United States; Vanderbilt University Medical Center, United States; Mayo Clinic of Jacksonville, FL, United States; University of Utah Health, United States; General University Hospital Consortium of Valencia, Spain; Vanderbilt University Medical Center, United States

We commend Hara et al. for undertaking a complex and industry-independent randomized controlled trial (RCT) of spinal cord stimulation (SCS) for persistent spinal pain following lumbar surgery [1]. We also wish to celebrate the recent publication by Dhurva et al. comparing SCS to conventional medical management [2]. However, we feel obligated to remark on methodological concerns and conclusions drawn from these publications to provide appropriate context and interpretation for providers, patients, and payers.

Hara et al. used a blinded design to compare sham stimulation to paresthesia-free stimulation described by the authors as “burst” stimulation. This “burst” stimulation was dissimilar to established SCS therapies and its efficacy has not been demonstrated [[3], [4], [5]]. Sham control is useful to assess the efficacy of this particular SCS setting, but inference cannot be made regarding any other stimulation parameter without additional research.

Another concern is that the SCS intervention in this study exclusively used one type of paresthesia-free stimulation, preventing optimization of the therapy based on individual responses to the full complement of available SCS settings, as per standard of care [6,7]. This element of study design led to patient selection that does not reflect SCS in practice. The findings therefore do not generalize to SCS standard of care, despite the authors’ assertion of such.

In contrast, De Andres et al. published the results of a prospective, blinded, and non-industry financed RCT on patients with Failed Back Surgery Syndrome demonstrated meaningful pain relief at conventional and high-frequency SCS settings [8]. The authors conclude that failed SCS at one setting or waveform does not translate to failed SCS with every waveform and encourage identifying the SCS setting(s) that lead to the best treatment outcomes for each patient [6,8].

Another recent article on SCS from Dhruva et al. retrospectively assessed opioid usage, interventional procedure usage, and cost following SCS implantation compared to a control cohort among patients with chronic pain. Their results revealed no statistically significant difference in opioid utilization and further spinal interventions 2 years after SCS placement compared to the control cohort[2]. These findings are in line with those published by Vu et al., showing no substantial reduction in opioid use after SCS implantation [9]. However, these results are dissimilar to previous publications which demonstrate varying levels of opiate reduction associated with SCS [[10], [11], [12]].

Moreover, we are not convinced that the cohorts are similar since they received dissimilar treatments. The supplemental data published along with the Dhruva et al. article paint a more complete picture. In the first year, SCS patients had a higher opioid dose than the non-SCS cohort, but similar in the second, which may indicate a decrease in opioid dose. Further, non-SCS patients were more likely to undergo spine surgery in the first year compared to the SCS cohort, which was not pointed out by the authors but may represent a very clinically significant outcome difference between the groups.

Finally, we must note that the outcomes reported (opioid dose and use of spinal injections) are worthwhile data points that may be useful for health systems and public health considerations but are not surrogates for the treatment's effects on pain or function. Thus, conclusions regarding efficacy in terms of pain and function should not be drawn.

Our hope is that these publications and our commentary may be used to guide future industry-independent studies to best evaluate the potential of SCS. We applaud efforts to provide real-world data as well as RCTs that are not sponsored by industry but encourage our colleagues against broad generalizations in their conclusions.

## Declaration of competing interest

The authors declare the following financial interests/personal relationships which may be considered as potential competing interests:Byron Schneider reports a relationship with Spine Intervention Society that includes: board membership and travel reimbursement. Zach McCormick reports a relationship with Spine Intervention Society that includes: board membership. Zach McCormick reports a relationship with 10.13039/100008497Boston Scientific Corporation that includes: funding grants. Ad hoc editor for Interventional Pain Medicine - Byron Schneider Associate Editor, Biologics - Regenerative Medicine for Interventional Pain Medicine - Christine Hunt Associate Editor, Minimally Invasive Surgery for Interventional Pain Medicine - W. Evan Rivers.
